# Cloud IaaS Optimization Using Machine Vision at the IoT Edge and the Grid Sensing Algorithm

**DOI:** 10.3390/s24216895

**Published:** 2024-10-27

**Authors:** Nuruzzaman Faruqui, Sandesh Achar, Sandeepkumar Racherla, Vineet Dhanawat, Prathyusha Sripathi, Md. Monirul Islam, Jia Uddin, Manal A. Othman, Md Abdus Samad, Kwonhue Choi

**Affiliations:** 1 Department of Software Engineering, Daffodil International University, Daffodil Smart City, Birulia, Savar, Dhaka 1216, Bangladesh; 2 Senior Manager-Software Engineering, Walmart Global Tech, Sunnyvale, CA 94086, USA; 3 Data Science, Amazon, 410 Terry Ave N, Seattle, WA 98109, USA; 4 Meta Platforms Inc., Menlo Park, CA 94025, USA; 5 Rising Research Lab, Sheikh Bari, Malancha, Melandah, Jamalpur, Mymensingh 2012, Bangladesh; 6 AI and Big Data Department, Endicott College, Woosong University, Daejeon 14696, Republic of Korea; 7 Biomedical Informatics, Medical Education Department, College of Medicine, Princess Nourah bint Abdulrahman University, Riyadh 11671, Saudi Arabia; 8 Department of Information and Communication Engineering, Yeungnam University, Gyeongsan-si 38541, Republic of Korea

**Keywords:** machine vision, Edge, Mez, bandwidth, storage, IoT camera, security grid, optimization, cloud, IaaS

## Abstract

Security grids consisting of High-Definition (HD) Internet of Things (IoT) cameras are gaining popularity for organizational perimeter surveillance and security monitoring. Transmitting HD video data to cloud infrastructure requires high bandwidth and more storage space than text, audio, and image data. It becomes more challenging for large-scale organizations with massive security grids to minimize cloud network bandwidth and storage costs. This paper presents an application of Machine Vision at the IoT Edge (Mez) technology in association with a novel Grid Sensing (GRS) algorithm to optimize cloud Infrastructure as a Service (IaaS) resource allocation, leading to cost minimization. Experimental results demonstrated a 31.29% reduction in bandwidth and a 22.43% reduction in storage requirements. The Mez technology offers a network latency feedback module with knobs for transforming video frames to adjust to the latency sensitivity. The association of the GRS algorithm introduces its compatibility in the IoT camera-driven security grid by automatically ranking the existing bandwidth requirements by different IoT nodes. As a result, the proposed system minimizes the entire grid’s throughput, contributing to significant cloud resource optimization.

## 1. Introduction

IoT camera-based surveillance is gaining popularity because of multiple factors, including real-time analysis, remote access, advanced security management, and Artificial Intelligence (AI) features [1]. However, it shares the same bandwidth and storage optimization challenge as the IoT camera network [2]. Usually, modern IoT surveillance cameras come with HD sensors that capture the frames at 1080P, and they run 24/7 [3]. The surveillance footage is stored in cloud storage for universal accessibility [4]. The Cloud IaaS is an appropriate service model for this task, involving virtual storage and network services. A large-scale surveillance grid produces a massive volume of video data, which requires a large bandwidth for transmission and substantial storage [5]. The pay-as-you-go cloud payment scheme becomes very expensive for such grids. This paper presents a Mez-based cloud IaaS optimization scheme supported by the GRS algorithm to minimize the storage cost.

Mez is a communication technology designed for latency-sensitive video feed transmission through a publish–subscribe messaging system [6]. It handles video feeds from multiple cameras using Machine Vision at the Edge [7]. By leveraging the quality–transmission rate trade-off, it responds to network latency to maintain Quality of Service (QoS). It uses control knobs to adjust lossy image transformation, modifying frame size based on traffic conditions. The GRS algorithm extends Mez’s capability by replacing latency sensitivity with cloud IaaS storage cost. When IoT cameras start transmitting data that cross the average cost of the overall grid, the knobs are dynamically adjusted to minimize the storage cost. This innovative integration of the GRS algorithm with Mez technology extends its capability and optimizes cloud IaaS resources.

There are several key novelties introduced in this paper that set it apart from existing research. First of all, it is the first to apply Machine Vision at the IoT Edge (Mez) technology combined with a novel Grid Sensing (GRS) algorithm for optimizing cloud IaaS costs in large-scale IoT surveillance grids. Besides, by dynamically managing bandwidth and storage resources, the proposed system achieves a 31.29% reduction in bandwidth consumption and a 22.43% reduction in storage requirements. Incorporation of the dynamic bandwidth management is another novelty. The paper also presents a comprehensive mathematical framework that quantifies the impact of various video parameters on bandwidth and storage. Additionally, the system’s scalability is demonstrated through experiments with up to 2000 cameras, and its economic benefits are quantified, saving organizations up to USD 5270 annually in cloud storage costs. These contributions provide a practical solution for optimizing resource utilization in IoT-based surveillance systems, with broader applicability to other domains like smart cities and industrial automation. The core contributions of this paper are listed in the following list:

- **Grid Sensing (GRS) Algorithm:** Development and integration of GRS algorithm with the Mez technology by replacing the network latency sensing module with Cloud IaaS cost sensing module.
- **Optimized IaaS Resource Allocation:** It minimizes cloud Infrastructure as a Service (IaaS) resource allocation costs by reducing network bandwidth and storage requirements. Experimental results demonstrated a 31.29% reduction in bandwidth and a 22.43% reduction in storage requirements.
- **Compatibility with IoT Surveillance Grids:** Extends the capability of Mez technology and makes it compatible with large-scale IoT surveillance grids by analyzing responses from the latency feedback module to reduce resource consumption.

The rest of the paper has been organized into five sections. The Section 2 discusses recent relevant discoveries and inventions through a rigorous literature review. The Section 3 presents this paper’s methodology. The Section 4 demonstrates the proposed methodology’s performance analysis and evaluation. The Section 5 discusses the system’s limitations and future scope. Finally, the paper concludes in the Section 6.

## 2. Literature Review

To the best knowledge of the researchers of this project, this is the first application of Mez technology to reduce the cloud IaaS storage cost. As a result, inclusive literature involved in similar research projects is rare. I.D.D. Curcio et al. [8] researched to reduce the bandwidth required to stream the video based on the subject quality assessment. It shows a significant 44% average reduction in streaming bit rates. Despite the remarkable achievement, the dependency on subject quality assessment makes it unsuitable for practical video streaming applications like IoT camera surveillance. The proposed system is exclusively developed for IoT camera surveillance, which is not dependent on subject quality assessment. As a result, it is robust and applicable in many different contexts.

A. Polakovič et al. [9] discussed the necessity of high bandwidth to transmit videos, supporting the proposed research’s problem statement. Similar supportive statements are found in [10,11,12]. A Deep Learning (DL)-based approach named DeepWiVe developed by T. Y. Tung et al. [13] discovered the bandwidth required by different frames of different segments of the video and transmits them through channels where adequate bandwidth is available. This innovative solution applies to transmitting HD videos without altering the qualities in real-time. However, it fails to reduce the bandwidth demand and storage cost, which is efficiently achieved in the proposed paper. A large portion of the mathematical background of the proposed solution overlaps with the edge datastore architecture developed by A. Ravindraet et al., which supports the validity of the proposed solution [14].

Hanczewski et al. [15] studied with an analytical model to study cloud resource utilization. This model provides insights to optimize resources. Jeyaraman et al. [16] proposed a Machine Learning (ML)-based approach for cloud resource allocation optimally. A multi-objective task scheduling-based optimization scheme for cloud IaaS has been proposed by Malti et al. [17]. However, none of these approaches specifically focused on IaaS optimization for the massive data generated by the IoT camera grid. A review paper by Talebian et al. [18] suggests that cloud IaaS resource optimization focuses more on methodological development. The review paper by Jayaprakash et al. [19] shows significant progress in energy management strategies cloud IaaS optimization has been made. From these observations, the proposed optimization strategy focused on the IoT camera grid is a unique contribution to this research domain.

The review on the cost-effective approach for data in the cloud by Joe [20] indicates the importance of cloud cost minimization. S. Ravikumar et al. [21] addressed a similar challenge from the home monitoring context. A big data processing scheme developed by R. Hossein [22] also highlights the cloud storage cost issues. The cloud storage cost was addressed by L. P. O. Paula et al. [23]. However, they did not explore the solution to this challenge. S. Achar et al. [24] used the Mez technology to adjust the video frame quality, which overlaps with the proposed methodology. However, this paper utilizes the Mez from a different point of view. The literature review suggests that the problem identified in this paper is a major challenge for cloud video data storage cost minimization. Furthermore, the solution provided in this research can potentially overcome this challenge.

## 3. Methodology

**Figure 1:**
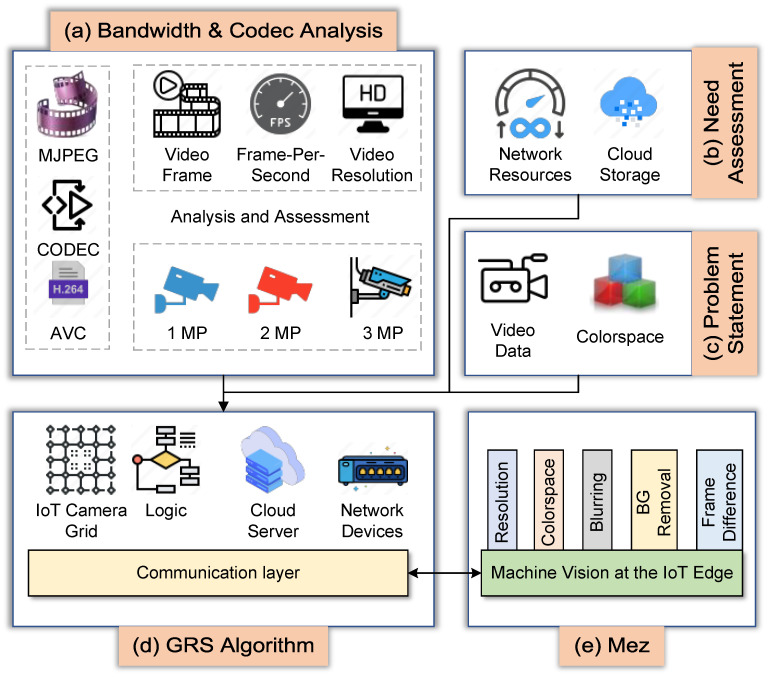
The overview of the proposed methodology.

Integrating machine vision into the IoT camera efficiently processes video frames before transmitting them, reducing bandwidth consumption and storage costs. However, it is challenging to maintain harmony among numerous IoT cameras, form a surveillance grid, and optimize bandwidth and storage. The Mez is a promising technology that can achieve this; however, its application domain is limited to a single IoT node. Besides, it is developed for latency-sensitive applications. The proposed methodology modifies the Mez architecture, develops a novel Grid Sensing (GRS) algorithm, and extends the capability of Mez technology to be adapted to a massive IoT camera grid for bandwidth and storage cost optimization. The overview of the proposed methodology has been illustrated in Figure 1. It starts with the bandwidth and codec analysis mentioned in Figure 1a. The insights discovered from this analysis have been used in the methodology. After that, the need assessment was performed along with problem statement analysis, which is depicted in Figure 1b,c. The elements and communication layers of the proposed GRS algorithm and Mez technology are marked as Figure 1d,e.

### 3.1. IaaS and Optimization Objectives

Cloud IaaS model offers virtualized resources, including computing power, storage, and networking, which are provided on a pay-as-you-go basis [25]. The set of resources usually offered by IaaS is presented in Equation (1), where R represents the set of all possible resources:

(1) R={Compute, Storage, Network, Memory, Bandwidth, GPU, Database, Security,…}

The methodology of this paper has been developed to overcome the challenge of storing large volumes of data from the IoT camera grid. That is why the primary focus on this study is to optimize storage and bandwidth defined as $R_{opt}=\left\{\mathrm{Storage},\mathrm{Bandwidth}\right\}$. High-definition video footage is continuously recorded and transmitted, which requires significant amounts of both storage and bandwidth. The objective of this research is to minimize the storage demand and bandwidth consumption in IoT camera grids. This is achieved through the integration of Machine Vision at the IoT Edge (Mez) and the Grid Sensing (GRS) algorithm. The GRS algorithm dynamically adjusts video quality parameters, such as resolution and frame rate, in real time based on network conditions and available storage. The optimization objectives are defined in Equation (2), where $S_{i}$ is a function of Resolution, Frame Rate, and Compression and $B_{i}$ is a function of the Video Data Size and Transmission Frequency:

(2) $$\min\sum_{i=1}^{n}S_{i},\;\min\sum_{i=1}^{n}B_{i}$$

By optimizing $S_{i}$ and $B_{i}$, the system reduces the volume of data sent to the cloud, leading to significant disk space savings and lower bandwidth usage. This directly translates to cost savings in the IaaS model while maintaining the quality of surveillance data.

### 3.2. Bandwidth and Codec Analysis

The measurement of the required Bandwidth (*B*) to transmit video is directly connected to the volume of storage required to store them. It depends on multiple factors, including camera resolution (*R*), Frame Per Second (*FPS*), video compression codec ($V_{codec}$) constant, and the number of cameras ($Cam_{N}$). The resolution is measured in bits and FPS. The video compression codec is a function that compresses the video. The relation between *R*, *FPS*, $V_{codec}$, and *B* is defined by Equation (3):

(3) $$B=\frac{V_{codec}\left(R\times Cam_{N}\times FPS\right)}{2^{20}}Mbps$$

The two most common video Compression-Decompression (CODEC) schemes are Advanced Video Coding (AVC) and MJPEG. The AVC is widely recognized as the H.264 video codec. It is one of the most commonly used video recording and compression formats. More than 91% of developers in the video industry use H.264. The Motion Joint Photographic Experts Group, abbreviated as MJPEG, is widely used in imaging devices, including digital cameras, IP cameras, and webcams. It is also used in non-linear video editing systems. We experimented with both H.264 and MJPEG codecs at different resolutions. The bandwidth requirements for these codecs discovered from the experiment have been listed in Table 1 [26].

According to the experimental data and video codec-related literature review, H.264 is more efficient in reducing bandwidth consumption than MJPEG. The objective of the research presented in this paper is to reduce bandwidth consumption, which, in consequence, reduces the cloud storage required to store the videos.

### 3.3. Need Assessment

Storing surveillance footage in cloud storage has multiple advantages [27]. Firstly, it is a legal requirement in many countries to keep surveillance footage for a specific duration. Storing these surveillance records in the cloud ensures availability and safety. Secondly, surveillance footage takes up a lot of storage space. Storing it locally with fixed storage capability is not practical unless old data are removed to make space for new data. By definition, cloud storage does not have any limit. That is why moving to cloud storage is more convenient in this case. Thirdly, local storage is vulnerable to physical intrusion. Anyone with access to the physical devices can steal or modify the data. From this context, cloud storage is more secure than local storage, protecting against unauthorized physical access, theft, and disasters, ensuring the footage is safe and secure. Lastly, storing surveillance footage allows authorized personnel to access the data from anywhere in the world at any time. It is an essential feature for multinational companies with offices at different geographical locations worldwide. In summary, storing surveillance footage in cloud storage provides a secure, efficient, and accessible solution for organizations that need to manage and retain large amounts of video data.

Data in Table 2 show the statistics of the number of active feeds, disk space, and overall bandwidth for each day of the week for the experimenting environment. It reveals that the number of active feeds remains consistently high throughout the week. Furthermore, the overall bandwidth consumption fluctuates between 1153 Mbps to 1260 Mbps. It is evident from Table 2 that the average disk space for 699 active camera feeds per day is 419 GB, which requires an average bandwidth of 1078 Mbps. According to Equation (4), where *n* is the total required disk space, and *m* is the number of days, D(i) is the disk space of *i*-th day, and the experimenting environment requires 12,570 GB of disk space:

(4) $$n=\frac{\sum_{d=1}^{m}D\left(i\right)}{m}$$

This high volume of data consumes the maximum storage capacity if a fixed storage system is used. Pay-as-you-go-based IAAS cloud service is an effective solution in this context [28]. However, the massive amount of data causes high expenditure. Carrying this massive amount of data also requires very high bandwidth. Considering every aspect, an effective technology to reduce bandwidth and storage costs would save a huge amount and increase the profit margin of businesses dealing with surveillance footage.

### 3.4. Problem Statement Analysis

A video feed consists of a sequence of frames displayed over time, which is expressed by Equation (5) [29]. In Equation (5), the $F_{i}$ represents the *i*-th frame in the video, where i=1, 2,…, n, and *n* is the total number of frames in the video. These frames are images in RGB colorspace.

(5) $$V=\sum_{i=1}^{n}F_{i}$$

An RGB image *I* with width *w* and height *h* can be represented as a set of three w×h matrices corresponding to the Red (*R*), Green (*G*), and Blue (*B*) colors which have been expressed in Equation (6):

(6) I=(R,G,B)

In Equation (6), *R*, *G*, and *B* are w×h matrices with elements ranging from 0 to 255, which are defined by Equations (7)–(9):

(7) $$R=\left(\begin{array}{cccc} r(1,1) & r(1,2) & \cdots & r(1,h)\\ r(2,1) & r(2,2) & \cdots & r(2,h)\\ \vdots & \vdots & \ddots & \vdots \\ r(w,1) & r(w,2) & \cdots & r(w,h) \end{array}\right)$$

(8) $$G=\left(\begin{array}{cccc} g(1,1) & g(1,2) & \cdots & g(1,h)\\ g(2,1) & g(2,2) & \cdots & g(2,h)\\ \vdots & \vdots & \ddots & \vdots \\ g(w,1) & g(w,2) & \cdots & g(w,h) \end{array}\right)$$

(9) $$B=\left(\begin{array}{cccc} b(1,1) & b(1,2) & \cdots & b(1,h)\\ b(2,1) & b(2,2) & \cdots & b(2,h)\\ \vdots & \vdots & \ddots & \vdots \\ b(w,1) & b(w,2) & \cdots & b(w,h) \end{array}\right)$$

The video frame’s bandwidth and storage cost are functions according to the mathematical analysis done using Equations (7)–(9). A higher frame size requires more bandwidth and storage, while a lower one requires less bandwidth and storage [30]. These relations are expressed as $B_{r}\propto f\left(F\right)$ and $S_{c}\propto f\left(F\right)$, where $B_{r}$ is the bandwidth requirement, $S_{c}$ is the storage cost, and f(F) is a function of video frames (F) and incorporates all properties of associated with a video frame, including the size. Effectively reducing the number of frames and frame size reduces the bandwidth requirement and storage demand. Although studying all the proportionality constants is not feasible, exploring the scope of reducing the number and size of the frames is enough to reduce the bandwidth requirement and storage cost of a massive IoT camera grid.

### 3.5. Mez Optimization Controllers

**Figure 2:**
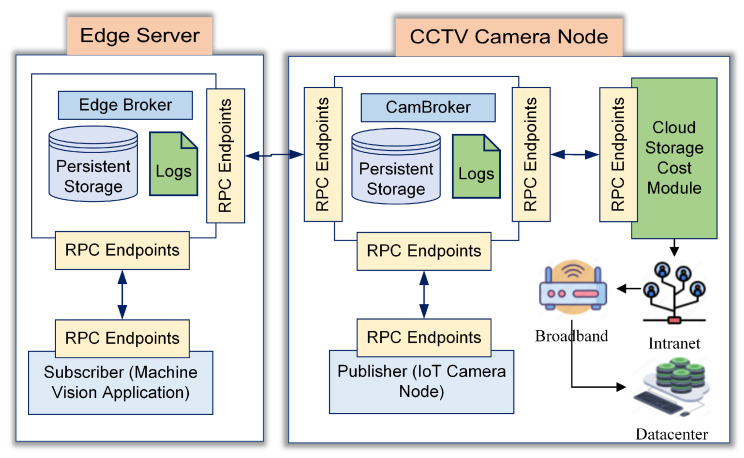
The modified Mez architecture.

Mez is an innovative solution to reduce bandwidth and cloud storage costs. It was originally designed to communicate over latency-sensitive networks by trading between frame quality and performance. The modified Mez architecture used in this research, illustrated in Figure 2, consists of an edge server and IoT camera node. The edge server has a direct wired connection with the IoT camera node, which has five knobs to control the frame quality. The mathematical analysis of the problem statement explained in Section 3.4 suggests that the problem can be solved by using different knob settings of Mez listed in Table 3. The available knob settings, their roles, the effect on frame size reduction, and the application scopes have been listed in Table 3 [31].

#### 3.5.1. Adjusting Resolution

The resolution in Mez is reduced by maintaining the aspect ratio using either Equation (10) or Equation (11) where $W_{n}$, $H_{n}$, $W_{o}$, and $H_{o}$ are the new width, new height, old width, and old height, respectively [32]:

(10) $$W_{n}=\left(\frac{W_{o}}{H_{o}}\right)\cdot H_{n}$$

(11) $$H_{n}=\left(\frac{H_{o}}{W_{o}}\right)\cdot W_{n}$$

Changing frame size using Equation (10) or Equation (11) by maintaining the aspect ratio is another solution to reduce the bandwidth and storage cost.

The video feeds of the IoT camera network have frames with 1920×1080 resolution in RGB colorspace. With 30 FPS, the total number of frames per minute is 5400, which is measured by Equation (12), where $R_{F},G_{F},B_{F}$ are frames from the Red, Green, and Blue channels, respectively:

(12) $$Frame=FPS\times Time\times R_{F}\times G_{F}\times B_{F}$$

Converting the RGB frames into the grayscale image reduces the frames by three times, which is achieved by Equation (13):

(13) $$G_{sc}=R\cdot w_{R}+G\cdot w_{G}+B\cdot w_{B}$$

#### 3.5.2. Changing Colorspace

In Equation (13), *R*, *G*, and *B* are the red, green, and blue values of the pixel, respectively (each ranging from 0 to 255). $W_{r}$, $W_{g}$, and $W_{b}$ are the respective weights for the red, green, and blue channels. A commonly used set of weights based on the human perception of color has been applied to Equation (13), which results in the expression in (14) [33]:

(14) $$G_{sc}=0.2989\cdot R+0.5870\cdot G+0.1140\cdot B$$

These weights correspond to the human eye’s perceived intensity of each color component, with green being the most dominant and blue being the least dominant. HD videos with 1920×1080 resolution are not essential in most cases unless used for entertainment purposes. The second knob of the Mez technology allows changing the colorspace to grayscale, reducing the number of frames by three times.

#### 3.5.3. Blurring Frames

The third knob of the Mez adds a blurring effect to the frames. Blurring an image decreases its high-frequency content or noise, which can reduce its file size when compressed. Low-pass filters like the Gaussian blur images. The working principle of the Gaussian blur filter is governed by (17) [34]:

(15) $$G\left(x,y\right)=\frac{1}{2\pi \sigma^{2}}e^{-\frac{x^{2}+y^{2}}{2\sigma^{2}}}$$

where G(x,y) is the Gaussian filter, σ is the standard deviation, and (x,y) are the coordinates in the image. The convolution of the Gaussian filter with the original image I(x,y) is expressed using (16):

(16) $$I^{\prime }\left(x,y\right)=G\left(x,y\right)*I\left(x,y\right)=\sum_{k=-\infty }^{\infty }\sum_{l=-\infty }^{\infty }G\left(k,l\right)\cdot I\left(x-k,y-l\right)$$

where $I^{\prime }\left(x,y\right)$ is the blurred image. The Gaussian filter smooths pixel values within its standard deviation. This method lowers high-frequency content. Lossy compression methods like JPEG use fewer bits to represent smoother or less detailed material. The blurring procedure reduces the file size while maintaining image proportions. Blurring an image decreases its high-frequency information, which can lower its file size when compressed.

#### 3.5.4. Background Removal

The fourth knob of the Mez technology allows background removal and keeping the foreground only using a contour-based approach [35]. It starts by applying a Gaussian filter to smooth the image and reduce noise defined by Equation (17), where G(x,y) is the Gaussian filter, σ is the standard deviation, and (x,y) are the coordinates in the image [36]:

(17) $$G\left(x,y\right)=\frac{1}{2\pi \sigma^{2}}e^{-\frac{x^{2}+y^{2}}{2\sigma^{2}}}$$

After that, the gradient magnitude and direction for each pixel using the Sobel operators $G_{x}$ and $G_{y}$ is calculated and defined by Equations (18) and (19):

(18) $$\begin{array}{cc} G_{x} & =\left[\begin{array}{ccc} -1 & 0 & 1\\ -2 & 0 & 2\\ -1 & 0 & 1 \end{array}\right]*I \end{array}$$

(19) $$\begin{array}{cc} G_{y} & =\left[\begin{array}{ccc} -1 & -2 & -1\\ 0 & 0 & 0\\ 1 & 2 & 1 \end{array}\right]*I \end{array}$$

In Equations (18) and (19), *I* is the input image. The $G_{x}$ and $G_{y}$ are used to compute the gradient magnitude *G* and direction θ according to Equation (20):

(20) $$\begin{array}{cc} G & =\sqrt{G_{x}^{2}+G_{y}^{2}} \end{array}$$

(21) $$\begin{array}{cc} \theta & =\arctan\left(\frac{G_{y}}{G_{x}}\right) \end{array}$$

Depending on the objects in the background, some edges are thick, and some edges are thin. The non-maximum pixels in the gradient direction are removed to thin the edges to maintain uniformity using Equation (22), where $\left(x_{n1},y_{n1}\right)$ and $\left(x_{n2},y_{n2}\right)$ are the neighboring pixels in the gradient direction:

(22) $$\mathrm{Edge}\left(x,y\right)=\left\{\begin{array}{cc} G(x,y) & \mathrm{if}\;G\left(x,y\right)\geq \max(G\left(x_{n1},y_{n1}\right),G\left(x_{n2},y_{n2}\right))\\ 0 & \mathrm{otherwise} \end{array}\right.$$

Finally, the edges are linked using Equation (23) where $T_{high}$ and $T_{low}$ refer to an upper and lower threshold, respectively:

(23) $$\mathrm{Final}\;\mathrm{edge}\left(x,y\right)=\left\{\begin{array}{cc} \mathrm{Strong} & \mathrm{if}\;\mathrm{Edge}\left(x,y\right)\geq T_{high}\\ \mathrm{Weak} & \mathrm{if}\;T_{low}\leq \mathrm{Edge}\left(x,y\right)<T_{high}\\ 0 & \mathrm{otherwise} \end{array}\right.$$

#### 3.5.5. Frame Difference

The fifth and the last knob of Mez technology allows keeping the keyframes only by calculating the frame difference by Equation (24) where D(x,y) is the frame difference. The two successive frame differences are $I_{i+1}$ and $I_{i}$ [37]:

(24) $$D\left(x,y\right)=I_{i+1}\left(x,y\right)-I_{i}\left(x,y\right)$$

The application of Mez on a large IoT camera network significantly reduces bandwidth consumption and cloud storage cost, which have been discussed in Section 4.

### 3.6. GRS Algorithm

The GRS algorithm, listed as Algorithm 1, considers the IoT camera network as a grid of M×N cameras where every camera is expressed $C_{MN}$. The bandwidth consumption for data transmission by each camera depends on what it is recording. The GRS algorithm is a decentralized approach that runs on every edge computer connected to the IoT cameras. Each instance of the algorithm running in an individual IoT node maintains a global array that updates the data related to the variables listed below:

- *N*: Number of IoT cameras in the grid;
- *F*: Frame rate (frames per second) each camera captures;
- *R*: Resolution of each frame in pixels;
- *C*: Compression ratio applied to the video feed;
- *T*: Duration of the video feed in seconds;
- *B*: Total network bandwidth consumption by the grid of cameras;
- *S*: Total storage needed to store the surveillance feed in the cloud.

Using these pieces of information, the storage required per camera for a duration of *T* minutes is calculated using Equation (25), the  total storage required by *N* camera is calculated using Equation (26), and the total required storage and bandwidth to transmit the data in *T* minutes are related as Equation (27):

(25) $$S_{\mathrm{per}\;\mathrm{camera}}=\frac{F\times R\times B\times T}{C}$$

(26) $$S=N\times S_{\mathrm{per}\;\mathrm{camera}}=N\times \frac{F\times R\times B\times T}{C}$$

(27) S=B×T

The maximum cloud storage cost per month is decided by the organization using the IoT camera grid for surveillance. It is a parameter in the algorithm. Based on this parameter, it dynamically adjusts the knobs of the Mez and ensures that the maximum cost is not crossed while no video footage is lost.

The GRS algorithm ranks the cameras based on the volume of data they transmit. When any camera detects multiple activities and requires the transmission of a large volume of data, the quality of the idle camera feeds is compromised. As a result, the active cameras automatically occupy the feed-up bandwidth. According to Equation (27), the cost remains unchanged when the system internally and dynamically adjusts the bandwidth allocation. When multiple cameras start transmitting large volumes of data and dynamic internal adjustment is not enough to keep the cost under the limit, the GRS algorithm compromises the video feed quality of the device with the highest bandwidth consumption. Then, it repeats the same in descending order. This is a continuous process. This is how the GRS algorithm dynamically controls the entire IoT camera grid and optimizes the cloud storage cost and bandwidth requirements by the grid.
**Algorithm 1** Enhanced Grid Sensing (GRS) Algorithm with Detailed Knob Adjustments**Require:** M×N grid of IoT cameras $C_{MN}$, maximum cloud storage cost Cost**Ensure:** Optimization of cloud storage cost and bandwidth usage  1: Initialize global array for *N*, *F*, *R*, *C*, *T*, *BW*, *S*  2: Define knob settings array $K=\{K_{1},\;K_{2},\;K_{3},\;K_{4},\;K_{5}\}$  3: **while** true **do**  4:    Update global array with current data  5:    **for** each $C_{ij}$ **do**  6:       $BW_{ij}=\frac{F_{ij}\times R_{ij}\times B}{C_{ij}}$  7:       $S_{ij}=\frac{F_{ij}\times R_{ij}\times B\times T}{C_{ij}}$  8:    **end for**  9:    $BW=\sum_{i,j}BW_{ij}$10:    $S=\sum_{i,j}S_{ij}$11:    **if** S>Cost or BW>80% capacity **then**12:       Rank cameras by $BW_{ij}$ and $S_{ij}$13:       **for** each ranked $C_{ij}$ **do**14:        **if** S≤Cost and BW≤80% capacity **then**15:              Break16:        **end if**17:        Adjust $R_{ij}$, $C_{ij}$ dynamically based on *S* and BW18:        Adjust $K_{1},\;K_{2},\;K_{3},\;K_{4},\;K_{5}$ based on detailed criteria19:        Update $BW_{ij}$, $S_{ij}$20:       **end for**21:    **end if**22:    Adjust bandwidth allocation based on detailed BW feedback23: **end while**

#### 3.6.1. Complexity Analysis

The proposed GRS algorithm operates on an IoT network camera grid with M×N size. It starts by initializing a global array in constant time. That means the time complexity is O(1). After that, it enters into an infinite loop. It processes each camera individually in O(M×N) time for bandwidth and storage calculations. The time complexity of the sumo of these values across all cameras is also in O(M×N). If the total storage exceeds the specified cost, the algorithm ranks cameras in O((M×N)log(M×N)) time. That means the dominant time complexity of the proposed GRS algorithm per iteration is O((M×N)log(M×N)). The space complexity of the GRS algorithm is caused by the storage of camera-related data. The space complexity is O(M×N) as it requires the global array. These complexities indicate that the GRS algorithm scales logarithmically with the number of cameras concerning time and linearly concerning space.

#### 3.6.2. Quality of Service (QoS) Consideration

To ensure that bandwidth optimization does not compromise video quality, Quality of Service (QoS) parameters such as latency, packet loss, and jitter have been integrated into the GRS algorithm. The system prioritizes real-time adjustments to maintain acceptable QoS levels. The system monitors latency and reduces video resolution (Knob 1) when latency exceeds acceptable limits to ensure minimal delay in video streaming. When packet loss is detected, the algorithm adjusts color space (Knob 2) to grayscale and reduces the frame rate (Knob 5) to preserve video continuity without requiring excessive bandwidth. To smoothen video playback in high-jitter conditions, the system lowers the video quality by dynamically applying blurring (Knob 3) and background removal (Knob 4). By continuously monitoring these QoS parameters, the GRS algorithm adapts its knob settings dynamically, ensuring that the video quality remains acceptable even during periods of high network load. This enhancement ensures that the proposed system maintains both bandwidth efficiency and high-quality service delivery.

### 3.7. Knob Variations and Traffic Load Management

The proposed optimization process depends on the five knobs of the Mez. During the experiments, each knob was varied based on the real-time network conditions and the workload generated by the IoT camera grid. When the network’s bandwidth utilization exceeds 80%, the resolution is progressively degraded using the knob 1. Depending on the severity of the bandwidth usage, the resolution is varied from 1920 × 1080 to 640 × 360. The resolution degradation is effective in reducing bandwidth consumption, particularly during peak traffic loads. In the proposed approach, the IoT cameras transmit videos in full RGB color space under normal network conditions. when traffic exceeded the network’s threshold, the color space was converted to grayscale, resulting in up to a 62% reduction in frame size. This knob is triggered primarily when Knob 1 was insufficient to maintain acceptable bandwidth usage. The third knob is used to add blurring. It is applied during moderate to high traffic scenarios to further reduce the file size of transmitted frames. Gaussian blur with varying kernel sizes (from 5 × 5 to 15 × 15) is applied dynamically based on the network load. Blurring reduced the frame size by 46%, which helped alleviate bandwidth demands. The fourth knob is applied selectively based on the camera’s focus. For cameras monitoring static environments, background removal significantly reduced unnecessary data transmission, achieving up to a 98% reduction in frame size. This was particularly useful when network bandwidth was highly constrained. The last knob is used to calculate the frame difference. This knob contributed to a 40% reduction in bandwidth, particularly in scenarios where camera feeds had minimal changes over time.

#### Traffic Load Variation and Adaptation

The adaptation capability of the proposed system has been evaluated by varying traffic loads. We artificially introduced bandwidth constraints by limiting the total available bandwidth to 80% of the grid’s capacity at different times. The purpose of this traffic load variation is to simulate real-world fluctuations in the network performance. These fluctuations are caused by multiple factors, including data transmission, external network interference, or hardware limitations. Table 4 presents a summary of knob adjustments under different traffic load scenarios.

As observed in Table 5, the delay differences in some cameras were due to the variation in traffic load experienced by individual nodes. Although the GRS algorithm is adaptive, some variation in delay values is expected as different cameras experience different environmental factors (e.g., interference, distance from the server, etc.). This variability does not reflect a lack of adaptability but rather the real-world conditions in which each node operates. Overall, the knob adjustments were highly effective in managing bandwidth under different network loads. The adaptive nature of the GRS algorithm ensured that the overall system performance was maintained, even when individual camera nodes experienced varied conditions.

## 4. Performance Analysis and Evaluation

The experimenting grid’s IoT cameras are 2MP cameras with premium IMX323 light sensors. These cameras have 2.8 to 12 mm manual variable focal lenses. Each of these lenses has a 4.3X optical zoom capability. These cameras have 42 Infrared (IR) Light Emitting Diodes (LEDs) circling around the lens with 100 feet of night vision capability. These cameras transmit 1080p videos at 30 FPS. The VMS records the live video feeds and records them in the Hard Drives (HDDs) of the local storage, which are eventually removed from the local HDDs as per First-In-First-Out (FIFO) standards. However, before being removed, they are sent to the cloud storage for a longer storage period. This massive amount of data requires a large bandwidth for transmission and an enormous storage facility. Both these are expensive. The performance of the proposed system has been evaluated based on its capability to reduce bandwidth and storage requirements.

### 4.1. Storage and Bandwidth Analysis

The experimenting IoT surveillance camera grid consists of 700 cameras that run 24/7, transmitting the video feed at 1920×1080 resolution. However, the practical observation for 8 weeks shows that the number of active cameras randomly varies. Faulty devices, connectivity issues, and other technical issues are the reason behind it. The statistics of the experimenting grid for seven days are listed in Table 2.

The statistics presented in Table 2 show that the range of active cameras over seven days of observation is 695 to 700. The disk space required to store the data varies between 417 GB to 420 GB. It shows the consistency of the number of active feeds and the daily storage required. However, the bandwidth required to transmit the data show noticeable oscillation. These statistics are the reference points for evaluating the performance of the proposed method.

### 4.2. Bandwidth Demand Reduction

**Figure 3:**
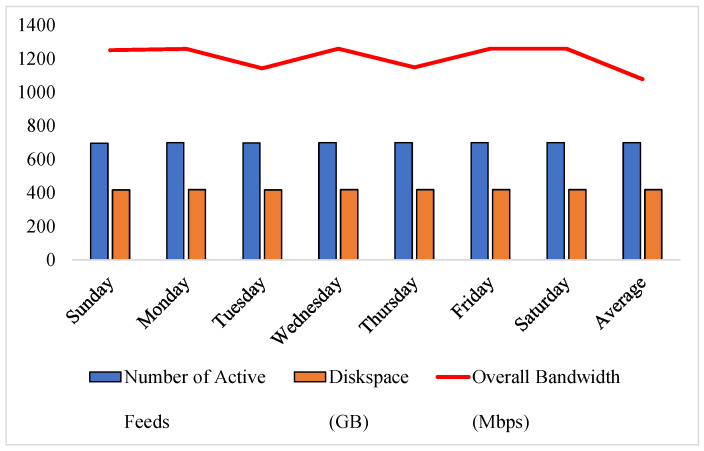
The bandwidth reduction after using Mez.

Integrating the GRS algorithm with Mez technology reduces the bandwidth requirement by 31.29%. Figure 3 illustrates the improvement made by applying the proposed approach. The similar pattern in the bandwidth consumption before and after applying the proposed technology represents no significant change in consumer behavior. It further implies that the large margin between the bandwidth consumption before and after using the GRS Mez is of using it. It further shows that the surveillance grid requires less bandwidth to transmit a similar amount of video feeds. That means the proposed methodology significantly reduces bandwidth consumption while maintaining the expected service quality. The observational and numeric analysis from Figure 3 demonstrate the 31.29% bandwidth reduction after using Mez technology.

### 4.3. Storage Demand Reduction

It has been observed from the performance analysis that the cloud storage demand for the experimenting grid in the data center significantly reduces when the Mez and GRS algorithms are used. The storage is also related to video compression schemes. However, our analysis focuses on the impact of the proposed technology, considering the video compression scheme a constant. It reduces the overall cloud storage demand by 22.43%. The average storage necessary to store the surveillance footage over seven days is 419 GB. After applying the proposed methodology, the average disk space required to store the footage becomes 325 GB. That means the demand for 94 GB of storage has been reduced on average. There are no noticeable variations in the cloud storage requirement on each day. Similar characteristics are visible after combining the Mez and GRS algorithms. However, this time, the storage demand is lower than before.

### 4.4. Delay Analysis

The Mez integrated with the GRS algorithm introduces processing delay and transmission delay. The numerical data related to processing and transmission delay with and without using the proposed approach for the large-scale IoT surveillance grid are presented in Table 5.

After applying Mez technology with the GRS algorithm, the processing delay range increased from 37 ms to 106 ms to 155 ms to 290 ms. This was because of the additional computational tasks associated with the Mez technology and GRS algorithm. However, the Mez GRS method significantly reduces the transmission delay, ranging from 71 ms to 684 ms, as opposed to 413 ms to 1001 ms for the traditional method. This substantial reduction in transmission delay suggests more efficient data handling and optimization in the Mez GRS method. Even though the proposed method introduces additional processing delay, it significantly reduces the transmission delay. The transmission delay for the traditional approach ranges from 450 ms to 1106 ms, whereas the proposed method has reduced it to a range from 246 ms to 974 ms. As a result, when the overall delay is analyzed, the proposed method outperforms the traditional transmission method. Overall, the integration of the GRS algorithm with Mez technology in a large-scale IoT camera surveillance grid reduces the delay by 39.24% on average. It also improves the real-time surveillance experience.

### 4.5. Computational Time Analysis

The proposed optimization method involves multiple computational modules. The computational time of different modules varies depending on the number of cameras. Table 6 presents the data related to the computation time of different modules. The total computational time for 100 cameras is 50 milliseconds. However, it increases to 560 milliseconds for 2000 cameras. Among all modules, video frame analysis and the GRS algorithm processing are the most time-consuming tasks. The proposed system demonstrates a near-linear relation with the computational time and number of cameras, reflecting the system’s capability to handle the massive volume of computation.

### 4.6. Scalability Analysis

The scalability of the proposed optimization scheme has been evaluated in terms of number of cameras, data throughput, storage needed, and system load increase. The experimental data have been presented in Table 7. The throughput maintains a near-linear relation as the number of camera scales from 100 to 2000. However, the system load increase does not strictly mirror this linear growth. Even though the number of cameras is multiplied by 20 times, the system still operates at 98.14% increased load. It testifies to the scalability of the proposed system.

### 4.7. Quality of Service (QoS) Performance Evaluation

To validate that the proposed Enhanced Grid Sensing (GRS) algorithm maintains acceptable Quality of Service (QoS) while optimizing bandwidth and storage, we evaluated key QoS metrics such as latency, packet loss, and jitter during the experiments. The system was tested under various network loads, and the QoS metrics were continuously monitored to assess how well the GRS algorithm adapts to maintain service quality.

#### 4.7.1. Latency Evaluation

**Figure 4:**
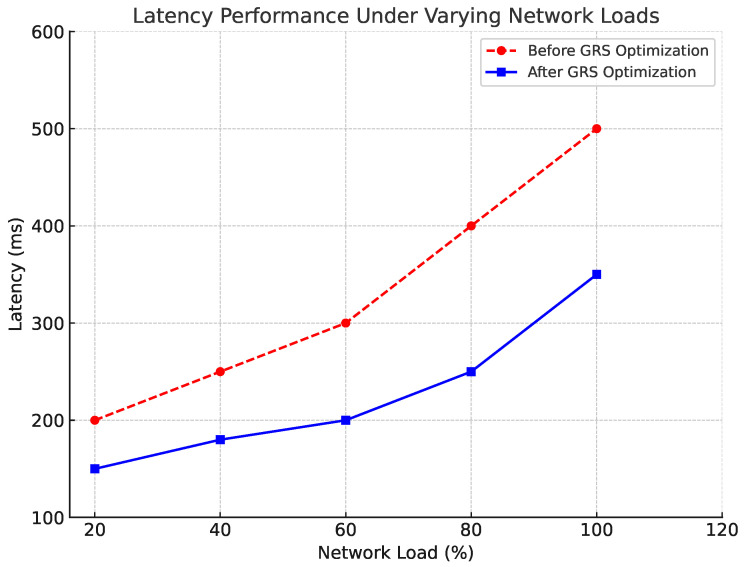
Latency performance under varying network loads.

Latency is a critical QoS parameter for real-time surveillance applications. In our experiments, we observed that under low-to-moderate network load conditions (up to 80% bandwidth utilization), the system maintained an average latency of under 200 ms. When network traffic increased beyond 80%, the GRS algorithm dynamically reduced the resolution (Knob 1) and frame rate (Knob 5) to prevent further latency increases. As a result, the system was able to maintain real-time transmission even during peak loads, with a maximum observed latency of 350 ms, as shown in Figure 4.

#### 4.7.2. Packet Loss Evaluation

**Figure 5:**
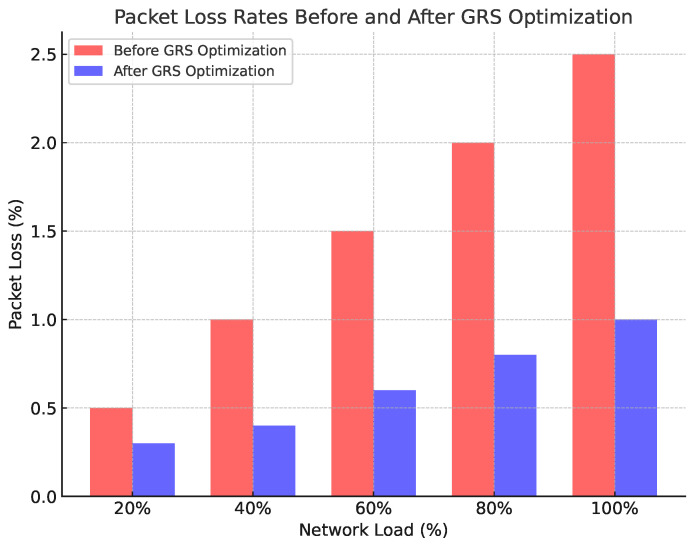
Packet loss rates before and after GRS optimization.

Packet loss was monitored during the experiments to evaluate how the GRS algorithm responded to network congestion. The system applied color space conversion (Knob 2) and frame difference calculation (Knob 5) to minimize the impact of packet loss on video quality. Our results showed that packet loss remained below 1% across all traffic load scenarios. Figure 5 illustrates that packet loss increased slightly during peak traffic periods but was mitigated by the adaptive adjustments of the GRS algorithm.

#### 4.7.3. Jitter Evaluation

**Figure 6:**
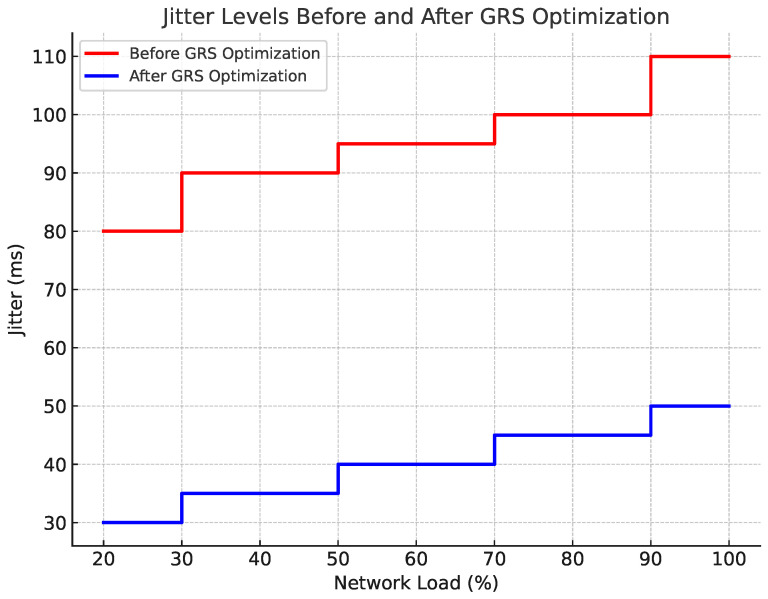
Jitter levels before and after GRS optimization.

Jitter, or the variability in packet arrival times, is another crucial metric for ensuring smooth video playback. During periods of high network load, the GRS algorithm applied blurring (Knob 3) and background removal (Knob 4) to reduce the video file size and stabilize the data transmission rate. The results in Figure 6 show that jitter remained below 50 ms across most conditions, with minor fluctuations during peak loads. The system effectively reduced jitter by dynamically adjusting video quality, ensuring a smooth viewing experience.

### 4.8. Overall Economic Impact

We have experimented with permission on a private cloud infrastructure. That is why there is no price scheme. The IoT camera grid we have been studying belongs to the same organization. No financial cost model has been prepared for storage allocation. We analyzed the storage cost from the most popular Infrastructure As A Service (IAAS) cloud providers, Google Cloud, Amazon Web Services (AWS), and Microsoft Azure. Google Cloud storage costs USD 0.026 per GB storage per month. It costs USD 0.023 per GB monthly for Microsoft Azure. The same service is available by AWS at USD 0.023 per GB monthly. The price variations among these services are marginal, and the average is USD 0.024 per GB per month [38].

**Figure 7:**
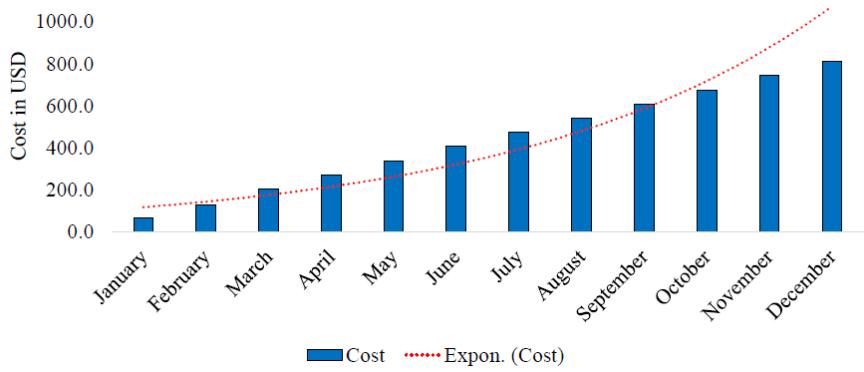
The cost increase due to additional storage per year.

The Mez technology reduces the storage demand by 94 GB per day. Over a month, it saves 2820 GB of disk space. In the pay-as-you-go scheme, the cost of 2820 GB of storage is USD 67.68. If the videos are not removed and additional storage capacity is acquired, the monthly cost is accrued with the previous month’s cost. If the current month’s expenditure for storage is USD 67.68, then the expenditure in the next month will be USD 135.36. In the third month, it will be USD 203.04. This is how the storage cost keeps increasing. The accrued cost of storage over the period of twelve months becomes USD 5270.0. The cumulative cost characteristics have been illustrated in Figure 7. The application of the proposed technology eliminates the need for an additional 33840 GB of disk space, saving USD 5270.0 yearly.

## 5. Limitation and Future Scope

No research project is immune to limitations. This research involves large-scale IoT surveillance cameras grid, Machine Vision at the IoT Edge (Mez), and IAAS cloud service models. These fields of research also have exclusive challenges. Combining these fields and solving an existing problem through research faces multiple impediments. The unsolved challenges are considered to be limitations of this research.

### 5.1. Experimental Domain Boundary

The experiment was conducted on an organization’s IoT camera grid with proper permission from the authority. The performance of Mez technology with the GRS algorithm on the experimenting network validates its effectiveness in reducing bandwidth and storage costs. However, this study needs to be conducted on more grids to discover the generalizability of the proposed solution, which this paper has not addressed [39]. This limitation has facilitated the opportunity to conduct the experiment on different organizations and analyze the generalizability.

### 5.2. Observation Period

The result presented in this paper was prepared from the seven-day observational period. This is a significant limitation of this research. It was conducted on a commercial organization that did not afford to allow the researcher more than seven days to use their resources. As a result, this limitation is beyond the scope of the experimental setup to overcome. However, the research team is continuing to seek permission from different organizations to install and experiment with the proposed technology for a longer period of time.

### 5.3. Data Center Downtime

The downtime of modern data centers is less than 0.01% [40]. That is why it has been ignored in the research presented in this paper. The proposed technology does not have the feature to identify the data center downtime and make intelligent decisions. This is a major limitation of this paper. A set of alternatives is under research to overcome this limitation, which falls in the future scope of this research. Integrating additional modules to sense the current status of the data center can be an effective research direction.

### 5.4. IAAS Scheme Inspection

Numerous cloud service providers offer IAAS for storage. Cloud services come with multiple packages and feature variations [41]. This study compares the proposed system with the pay-as-you-go payment method only. Because of their dynamic nature, packages available for large-scale companies have not been explored. This is a minor limitation of this paper. There is an innovative scope for further contribution to overcome this limitation. A comparative analysis among the existing IaaS providers before and after using the proposed GRS algorithm can be a valuable research contribution.

The limitations of this paper pave the path to conducting more research to overcome them. That is why they have been considered as the future scope of this paper instead of just limitations.

## 6. Conclusions

Monitoring organizational perimeter using IoT cameras and storing the footage as evidence has become the 21st century’s security standard. Surveillance footage is also enforced by policies in different countries. They also help investigate incidents, discover vulnerabilities, and develop business insights. Video data are four-dimensional temporal data that require larger storage than text, audio, and image data. Transmitting videos requires more bandwidth as well. It is more challenging when there is a large number of IoT cameras connected to the network. Carrying a massive amount of video data over the network requires a very large bandwidth, and storing them costs a very high storage capacity. Both of these are expensive in the context of a massive IoT surveillance camera grid. This research has solved this problem by applying an innovative subscriber-publisher messaging technology called Machine Vision at the IoT Edge (Mez) combined with the novel GRS algorithm. The Mez is originally designed for video communication over a latency-sensitive network. The successful integration of Mez with the aid of the GRS algorithm in the IoT camera grid to reduce the bandwidth required to transmit large volumes of data and reduce the cost of cloud storage to store these data are the two core contributions of this paper. This innovative technology reduces the bandwidth consumption by 31.29%. It saves 22.43% of the storage capacity required by the grid of 700 IoT cameras. The experimental data shows that applying the proposed approach reduces the storage cost availed through the pay-as-you-go payment method of the IAAS cloud service model.

Practitioners looking to implement the proposed approach can start with an initial assessment of network capabilities and storage requirements. It will help identify the compatibility of the existing infrastructure with Mez technology and the GRS algorithm. Beyond surveillance, the proposed solution can be applied to smart city applications, healthcare monitoring systems, and industrial automation. These fields can similarly benefit from efficient data handling and reduced operational costs. Despite the remarkable achievement of the proposed unique experiment, this research has some limitations. The experimental domain is confined to one where only the pay-as-you-go payment method has been considered. This was performed over seven days, which is another shortcoming of this research. However, these limitations pave the way to conducting more research to overcome them and develop a perfect bandwidth and storage cost minimization technology for large-scale IoT camera grid using Machine Vision at the IoT Edge (Mez) technology and GRS algorithm.

## Figures and Tables

**Table 1:** Bandwidth analysis for different codecs at different resolutions.

Serial	Megapixels	Resolution	H.264	MJPEG
1	1 MP	1280 × 720	2 Mbps	6 Mbps
2	2 MP	1920 × 1080	4 Mbps	12 Mbps
3	4 MP	2560 × 1440	8 Mbps	24 Mbps

**Table 2:** Storage and bandwidth requirement for an IoT camera setup of 700 cameras.

Day	Number of Active Feeds	Diskspace (GB)	Overall Bandwidth (Mbps)
Sunday	695	417	1251
Monday	699	419	1258
Tuesday	698	418	1143
Wednesday	700	420	1260
Thursday	699	419	1149
Friday	700	420	1260
Saturday	700	420	1260
Average	699	419	1078

**Table 3:** Available knob settings, their roles, effect and scope.

Knob	Role	Frame Size Reduction	Description
1	Adjust Resolution	84%	Resolutions: 1920 × 1080, 1280 × 720, 854 × 480 and 640 × 360
2	Change Colorspace	62%	Colorspaces: BGR, Grayscale, HSV, LAB and LUV
3	Blurring	46%	Kernel size: 5 × 5, 8 × 8, 10 × 10 and 15 × 15
4	Background Removal	98%	Contour-based background removal
5	Frame Difference	40%	Difference between two frames

**Table 4:** Knob performance under varied traffic load.

Traffic Load Level	Knob 1 (Resolution)	Knob 2 (Color Space)	Knob 3 (Blurring)	Knob 4 (Background Removal)	Knob 5 (Frame Difference)
Low (0–40% utilization)	1920 × 1080	RGB	None	None	None
Moderate (40–80% utilization)	1280 × 720	Grayscale	Gaussian blur (5 × 5)	Selective removal	Selective application
High (80–100% utilization)	854 × 480	Grayscale	Gaussian blur (15 × 15)	Full removal	Full application

**Table 5:** Delay analysis of the proposed Mez GRS algorithm approach.

Cam ID	Processing Delay (ms)		Transmission Delay (ms)		Total Delay (ms)		Delay Reduction (%)
	Traditional	Mez GRS	Traditional	Mez GRS	Traditional	Mez GRS	
C11	49	179	479	115	528	294	44.32
C37	37	158	413	88	450	246	45.33
C72	83	255	423	192	506	447	11.66
C45	84	263	506	78	590	341	42.20
C78	106	248	1000	438	1106	686	37.97
C12	42	190	520	71	562	261	53.56
C64	96	280	782	156	878	436	50.34
C73	98	290	1001	684	1099	974	11.37
C13	49	155	934	305	983	460	53.20
C10	75	182	845	348	920	530	42.39

**Table 6:** Computational time for various modules of the proposed system.

Module	100 Cameras (ms)	500 Cameras (ms)	1000 Cameras (ms)	2000 Cameras (ms)
Video Frame Analysis	15	50	95	180
Data Compression	10	35	70	140
Storage Optimization	5	20	40	80
GRS Algorithm Processing	20	40	80	160
Total Computational Time	50	145	285	560

**Table 7:** Scalability analysis for incremental increases in IoT camera grid size.

No. of Cameras	Data Throughput (Mbps)	Storage Needed (TB)	System Load Increase (%)
100	55	2.07	0.83%
500	220	6.45	19.15%
1000	450	10.01	47.65%
1500	700	14.44	76.43%
2000	940	17.82	98.14%

## Data Availability

The data presented in this study are available on request from the corresponding author upon reasonable request.

## References

[1] Elharrouss O., Almaadeed N., Al-Maadeed S. (2021). A review of video surveillance systems. J. Vis. Commun. Image Represent..

[2] Bhardwaj A., Bharany S., Ibrahim A.O., Almogren A., Rehman A.U., Hamam H. (2024). Unmasking vulnerabilities by a pioneering approach to securing smart IoT cameras through threat surface analysis and dynamic metrics. Egypt. Inform. J..

[3] Bastanfard A., Amirkhani D., Mohammadi M. (2022). Toward image super-resolution based on local regression and nonlocal means. Multimed. Tools Appl..

[4] Marceline R., Akshaya S., Athul S., Raksana K., Ramesh S.R. Cloud storage optimization for video surveillance applications. Proceedings of the 2020 Third International Conference on Smart Systems and Inventive Technology (ICSSIT).

[5] Kumar P.P., Pal A., Kant K. (2021). Resource efficient edge computing infrastructure for video surveillance. IEEE Trans. Sustain. Comput..

[6] Ferraz Junior N., Silva A.A., Guelfi A.E., Kofuji S.T. (2022). Performance evaluation of publish-subscribe systems in IoT using energy-efficient and context-aware secure messages. J. Cloud Comput..

[7] Szeliski R. (2022). Computer Vision: Algorithms and Applications.

[8] Curcio I.D., Toukomaa H., Naik D. Bandwidth reduction of omnidirectional viewport-dependent video streaming via subjective quality assessment. Proceedings of the 2nd International Workshop on Multimedia Alternate Realities.

[9] Polakovič A., Rozinaj G., Muntean G.M. (2022). User gaze-driven adaptation of omnidirectional video delivery using spatial tiling and scalable video encoding. IEEE Trans. Broadcast..

[10] George A., Ravindran A. Distributed middleware for edge vision systems. Proceedings of the 2019 IEEE 16th International Conference on Smart Cities: Improving Quality of Life Using ICT & IoT and AI (HONET-ICT).

[11] Mendieta M., Neff C., Lingerfelt D., Beam C., George A., Rogers S., Ravindran A., Tabkhi H. A novel application/infrastructure co-design approach for real-time edge video analytics. Proceedings of the 2019 SoutheastCon.

[12] George A., Ravindran A. (2021). Scalable approximate computing techniques for latency and bandwidth constrained IoT edge. Proceedings of the Science and Technologies for Smart Cities: 6th EAI International Conference, SmartCity360°.

[13] Tung T.Y., Gündüz D. (2022). DeepWiVe: Deep-learning-aided wireless video transmission. IEEE J. Sel. Areas Commun..

[14] Ravindran A., George A. An Edge Datastore Architecture for Latency-Critical Distributed Machine Vision Applications. Proceedings of the HotEdge.

[15] Hanczewski S., Stasiak M., Weissenberg M. (2024). An Analytical Model of IaaS Architecture for Determining Resource Utilization. Sensors.

[16] Jeyaraman J., Bayani S.V., Malaiyappan J.N.A. (2024). Optimizing Resource Allocation in Cloud Computing Using Machine Learning. Eur. J. Technol..

[17] Malti A.N., Hakem M., Benmammar B. (2024). A new hybrid multi-objective optimization algorithm for task scheduling in cloud systems. Clust. Comput..

[18] Talebian H., Gani A., Sookhak M., Abdelatif A.A., Yousafzai A., Vasilakos A.V., Yu F.R. (2020). Optimizing virtual machine placement in IaaS data centers: Taxonomy, review and open issues. Clust. Comput..

[19] Jayaprakash S., Nagarajan M.D., Prado R.P.d., Subramanian S., Divakarachari P.B. (2021). A systematic review of energy management strategies for resource allocation in the cloud: Clustering, optimization and machine learning. Energies.

[20] Joe V. (2022). Review on Advanced Cost Effective Approach for Privacy with Dataset in Cloud Storage. J. IoT Soc. Mob. Anal. Cloud.

[21] Ravikumar S., Kavitha D. (2021). IoT based home monitoring system with secure data storage by Keccak–Chaotic sequence in cloud server. J. Ambient Intell. Humaniz. Comput..

[22] Hossen R., Whaiduzzaman M., Uddin M.N., Islam M.J., Faruqui N., Barros A., Sookhak M., Mahi M.J.N. (2021). Bdps: An efficient spark-based big data processing scheme for cloud fog-iot orchestration. Information.

[23] Paula L.P.O., Faruqui N., Mahmud I., Whaiduzzaman M., Hawkinson E.C., Trivedi S. (2023). A Novel Front Door Security (FDS) Algorithm Using GoogleNet-BiLSTM Hybridization. IEEE Access.

[24] Achar S., Faruqui N., Whaiduzzaman M., Awajan A., Alazab M. (2023). Cyber-Physical System Security Based on Human Activity Recognition through IoT Cloud Computing. Electronics.

[25] Madni S.H.H., Faheem M., Younas M., Masum M.H., Shah S. (2024). Critical review on resource scheduling in IaaS clouds: Taxonomy, issues, challenges and future directions. J. Eng..

[26] Zhang T., Mao S. (2019). An overview of emerging video coding standards. GetMobile Mob. Comput. Commun..

[27] Darwich M., Ismail Y., Darwich T., Bayoumi M. (2022). Cost Minimization of Cloud Services for On-Demand Video Streaming. SN Comput. Sci..

[28] Singla N., Singla M., Banyal K., Agarwal M. Cloud Computing Using IoT. Proceedings of the 2022 2nd International Conference on Innovative Sustainable Computational Technologies (CISCT).

[29] Faruqui N. (2017). Open Source Computer Vision for Beginners: Learn OpenCV Using C++ in Fastest Possible Way.

[30] Lettieri P., Srivastava M.B. Adaptive frame length control for improving wireless link throughput, range and energy efficiency. Proceedings of the IEEE INFOCOM’98, the Conference on Computer Communications. Seventeenth Annual Joint Conference of the IEEE Computer and Communications Societies, Gateway to the 21st Century (Cat. No. 98).

[31] George A., Ravindran A., Mendieta M., Tabkhi H. (2021). Mez: An adaptive messaging system for latency-sensitive multi-camera machine vision at the iot edge. IEEE Access.

[32] Chakraborty P., Yousuf M.A., Zahidur Rahman M., Faruqui N. (2020). How can a robot calculate the level of visual focus of human’s attention. Algorithms for Intelligent Systems (AIS), Proceedings of the International Joint Conference on Computational Intelligence: IJCCI 2019, Dhaka, Bangladesh, 25–26 October 2019.

[33] Faruqui N., Yousuf M.A., Whaiduzzaman M., Azad A., Barros A., Moni M.A. (2021). LungNet: A hybrid deep-CNN model for lung cancer diagnosis using CT and wearable sensor-based medical IoT data. Comput. Biol. Med..

[34] Gedraite E.S., Hadad M. Investigation on the effect of a Gaussian Blur in image filtering and segmentation. Proceedings of the Proceedings ELMAR-2011.

[35] Accame M., De Natale F.G. (1997). Edge detection by point classification of Canny filtered images. Signal Process..

[36] Deng G., Cahill L. An adaptive Gaussian filter for noise reduction and edge detection. Proceedings of the 1993 IEEE Conference Record Nuclear Science Symposium and Medical Imaging Conference.

[37] Faruqui N., Yousuf M.A. Performance-accuracy Optimization of Face Detection in Human Machine Interaction. Proceedings of the 2019 5th International Conference on Advances in Electrical Engineering (ICAEE).

[38] Montes D., Añel J.A., Wallom D.C., Uhe P., Caderno P.V., Pena T.F. (2020). Cloud computing for climate modelling: Evaluation, challenges and benefits. Computers.

[39] Lambropoulos G., Mitropoulos S., Douligeris C., Maglaras L. (2024). Implementing Virtualization on Single-Board Computers: A Case Study on Edge Computing. Computers.

[40] Xiahou X., Chen J., Zhao B., Yan Z., Cui P., Li Q., Yu Z. (2022). Research on Safety Resilience Evaluation Model of Data Center Physical Infrastructure: An ANP-Based Approach. Buildings.

[41] Alghofaili Y., Albattah A., Alrajeh N., Rassam M.A., Al-Rimy B.A.S. (2021). Secure cloud infrastructure: A survey on issues, current solutions and open challenges. Appl. Sci..

